# Supplementary figures and images for: Crosstalk between repair pathways elicits double-strand breaks in alkylated DNA and implications for the action of temozolomide
Source: eLife. 2021 Jul 8;10:e69544. doi: 10.7554/eLife.69544 (PMC8289412; doi:10.7554/eLife.69544)

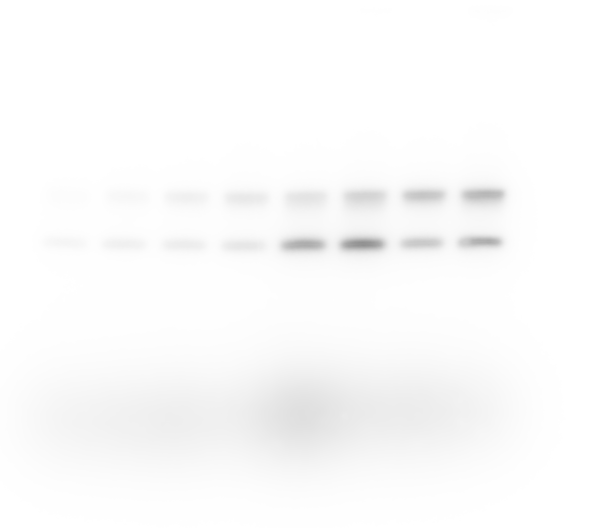

Supplement: Source data 1. [file elife-69544-data1.zip › eLife-source data/Figure 3-source dataC2.tif]

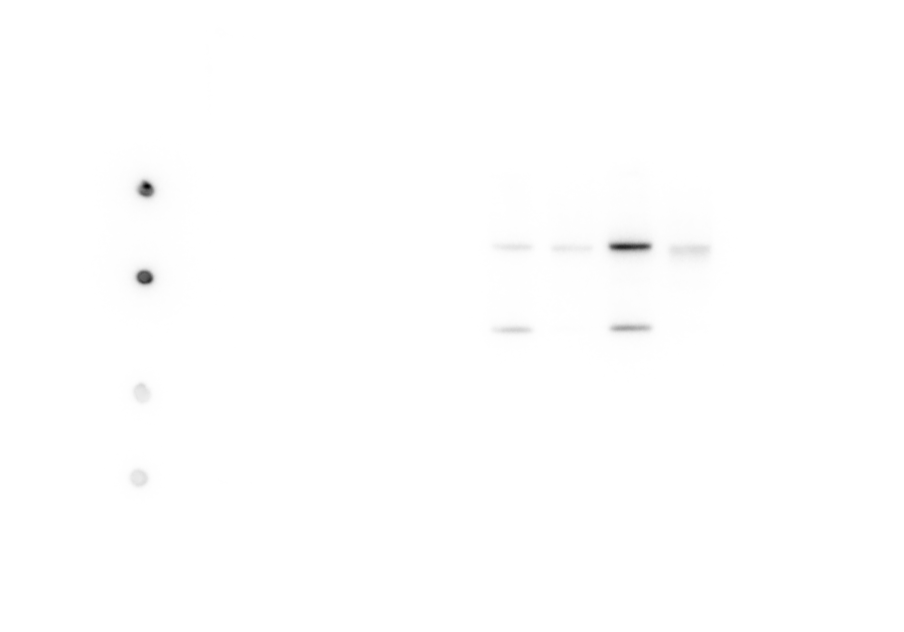

Supplement: Source data 1. [file elife-69544-data1.zip › eLife-source data/Figure 2-source dataC2.tif]

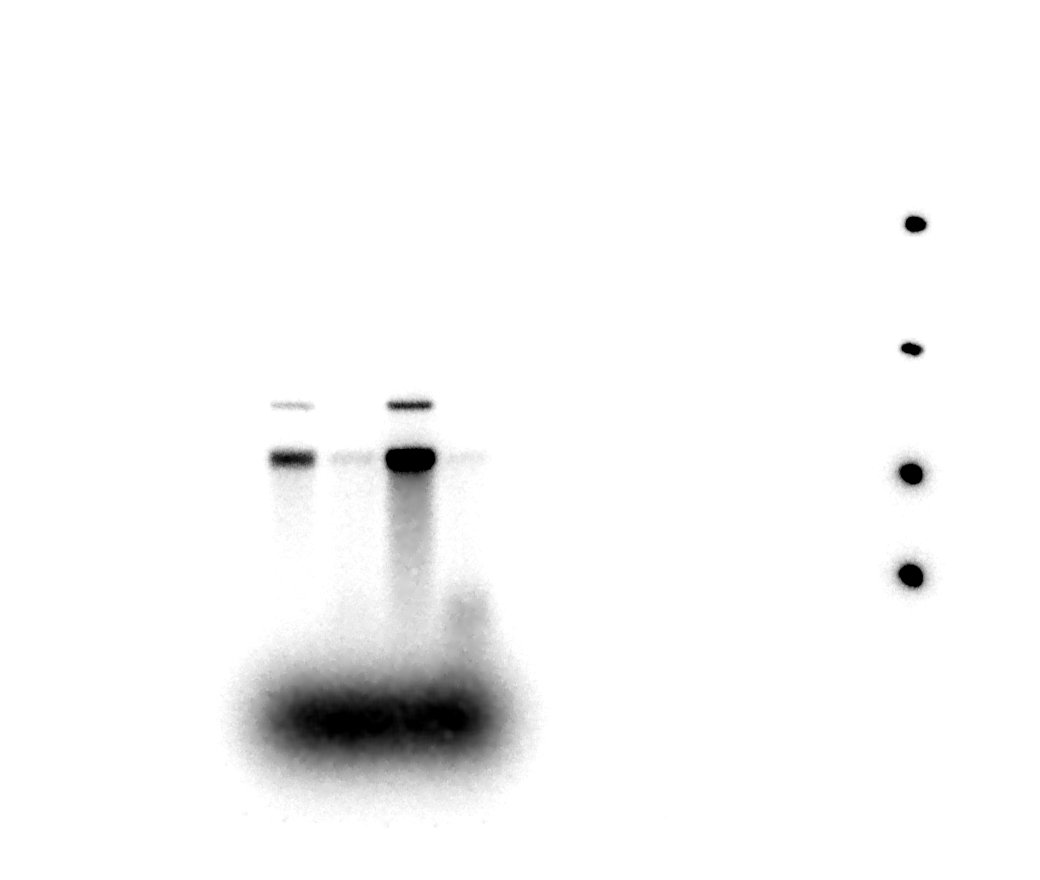

Supplement: Source data 1. [file elife-69544-data1.zip › eLife-source data/Figure 2-source dataD2.jpg]

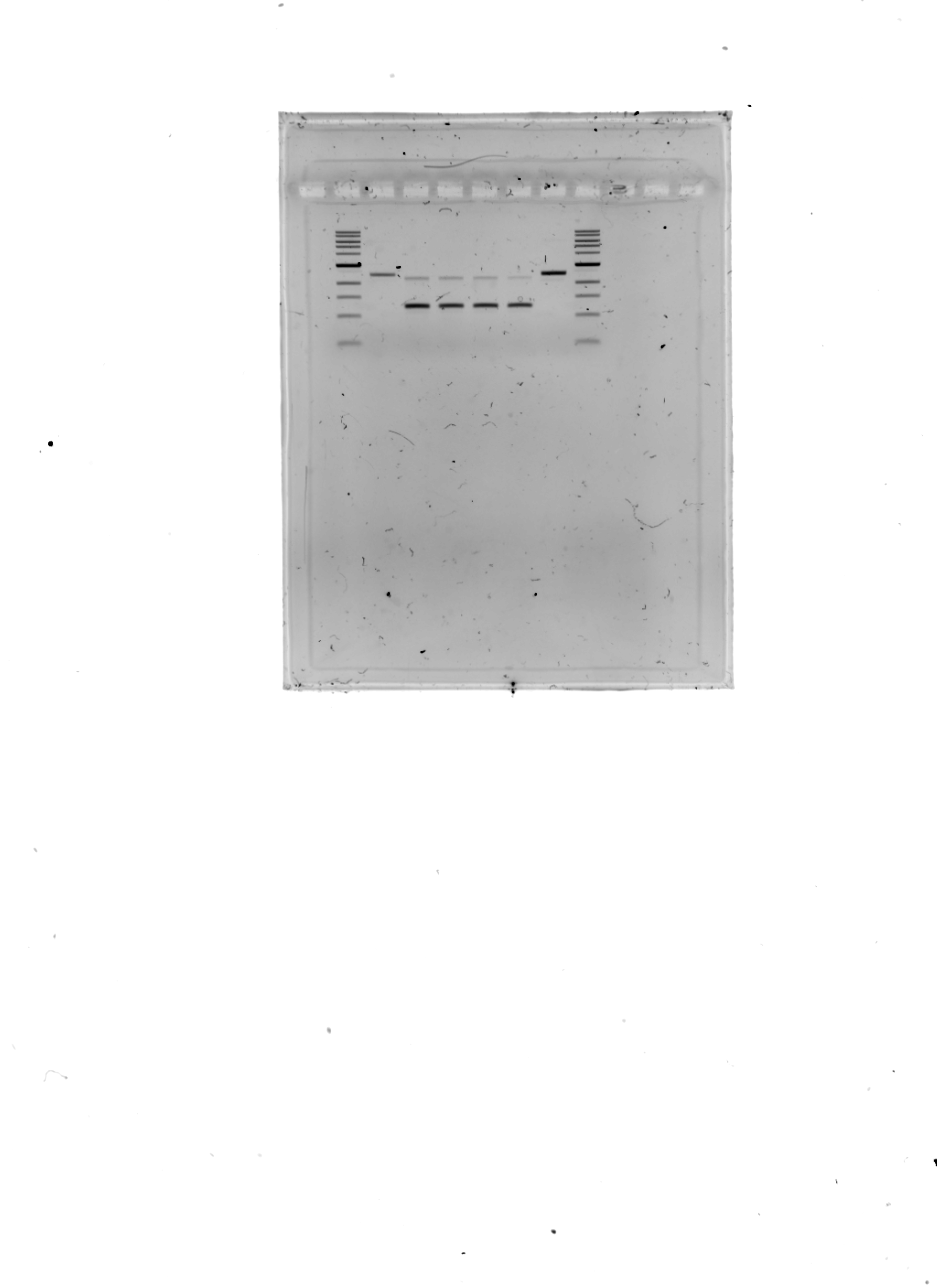

Supplement: Source data 1. [file elife-69544-data1.zip › eLife-source data/Figure 3-figure supplement1 source dataA.tif]

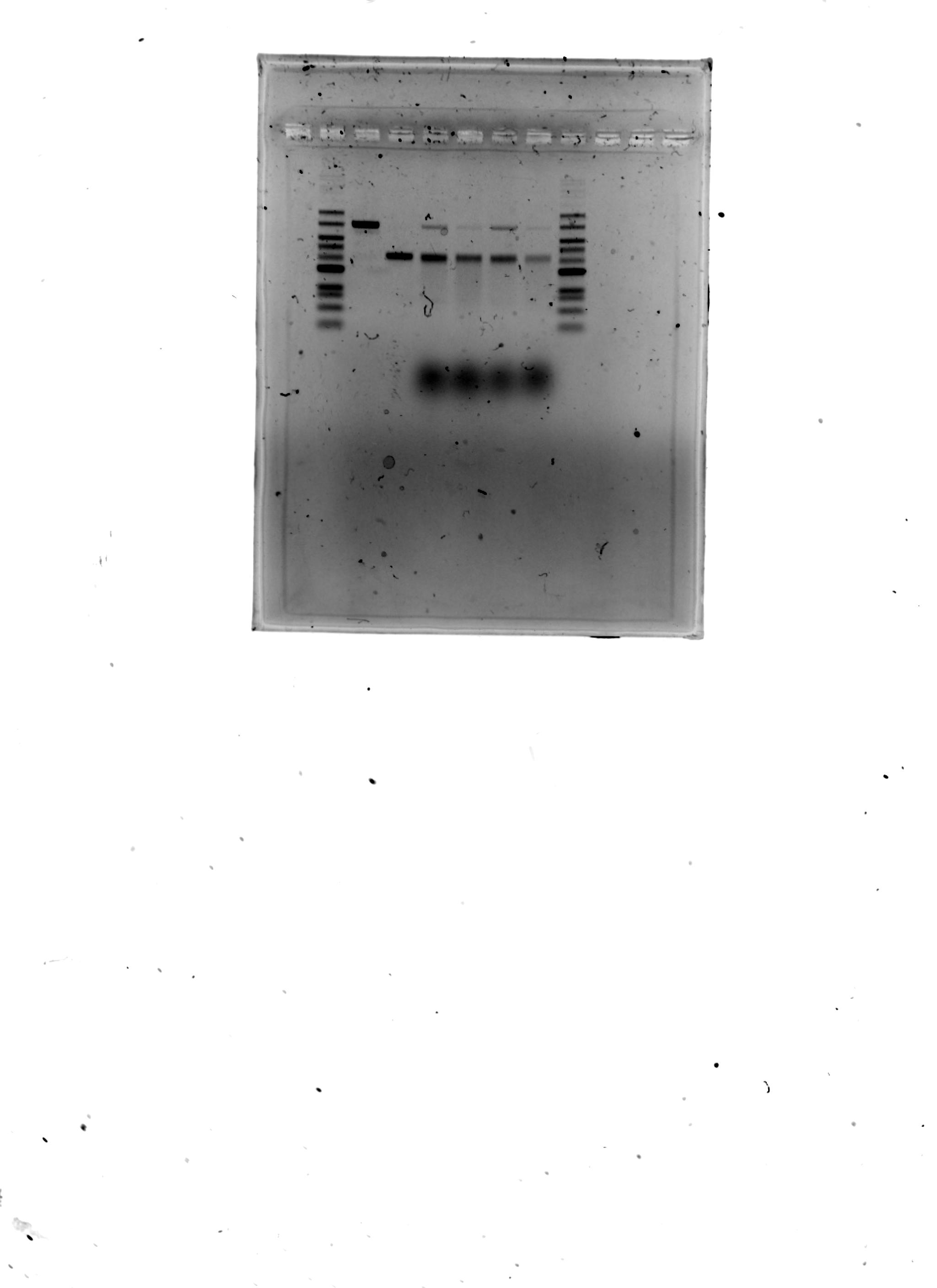

Supplement: Source data 1. [file elife-69544-data1.zip › eLife-source data/Figure 2-source dataD1.jpg]

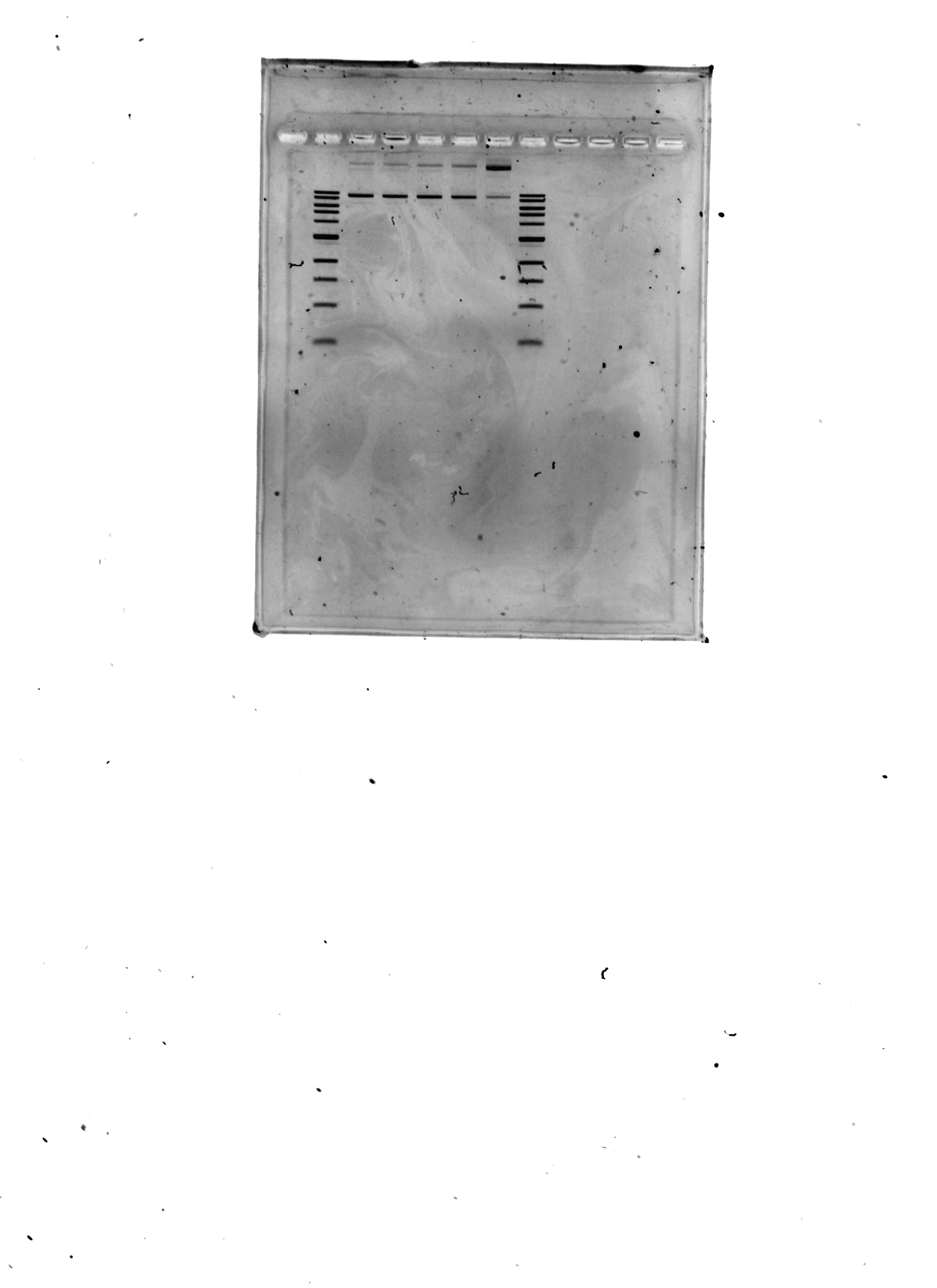

Supplement: Source data 1. [file elife-69544-data1.zip › eLife-source data/Figure 4-figure supplement2 source dataA.jpg]

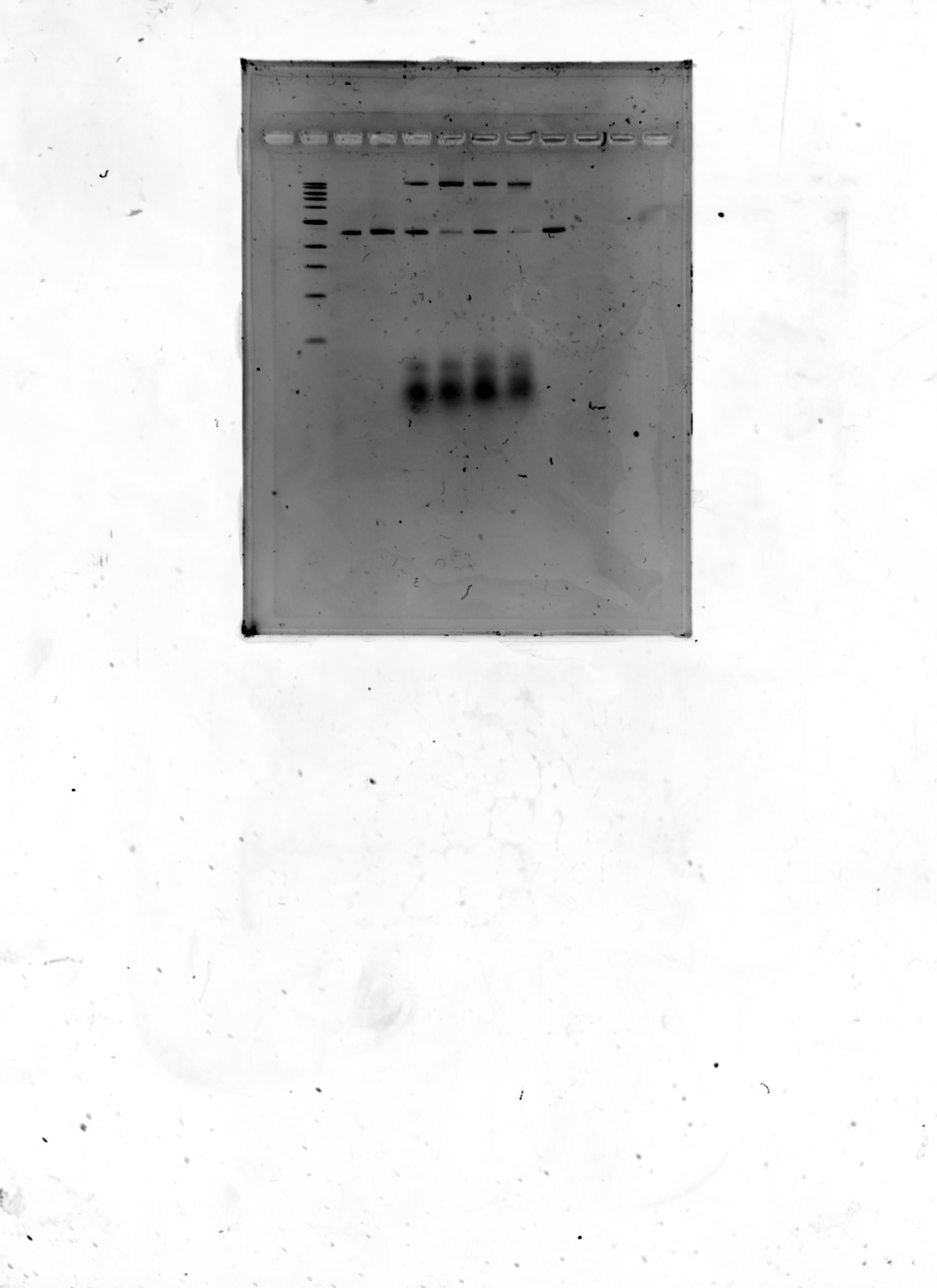

Supplement: Source data 1. [file elife-69544-data1.zip › eLife-source data/Figure 2-source dataC1.jpg]

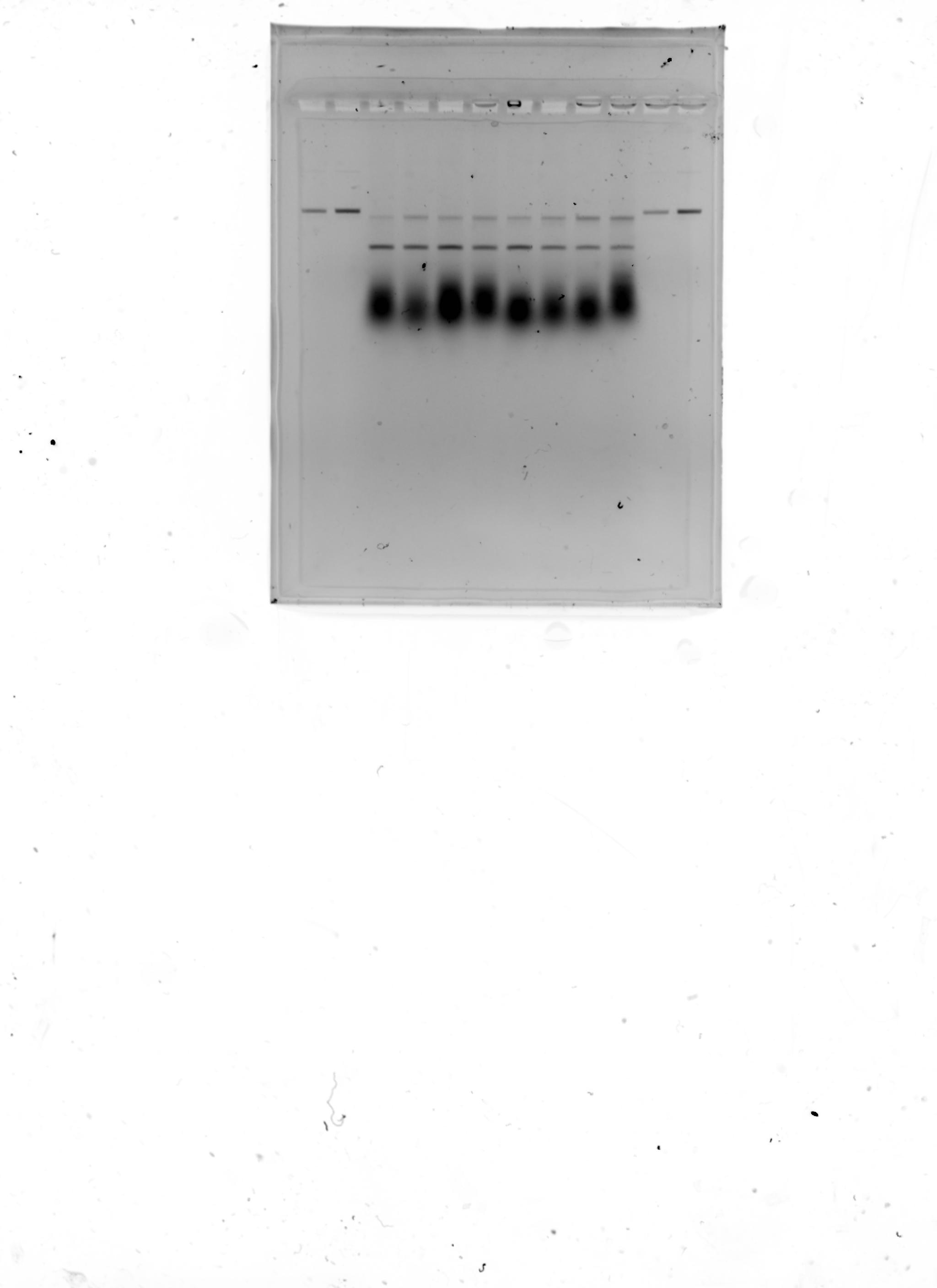

Supplement: Source data 1. [file elife-69544-data1.zip › eLife-source data/Figure 3-source dataC1.jpg]

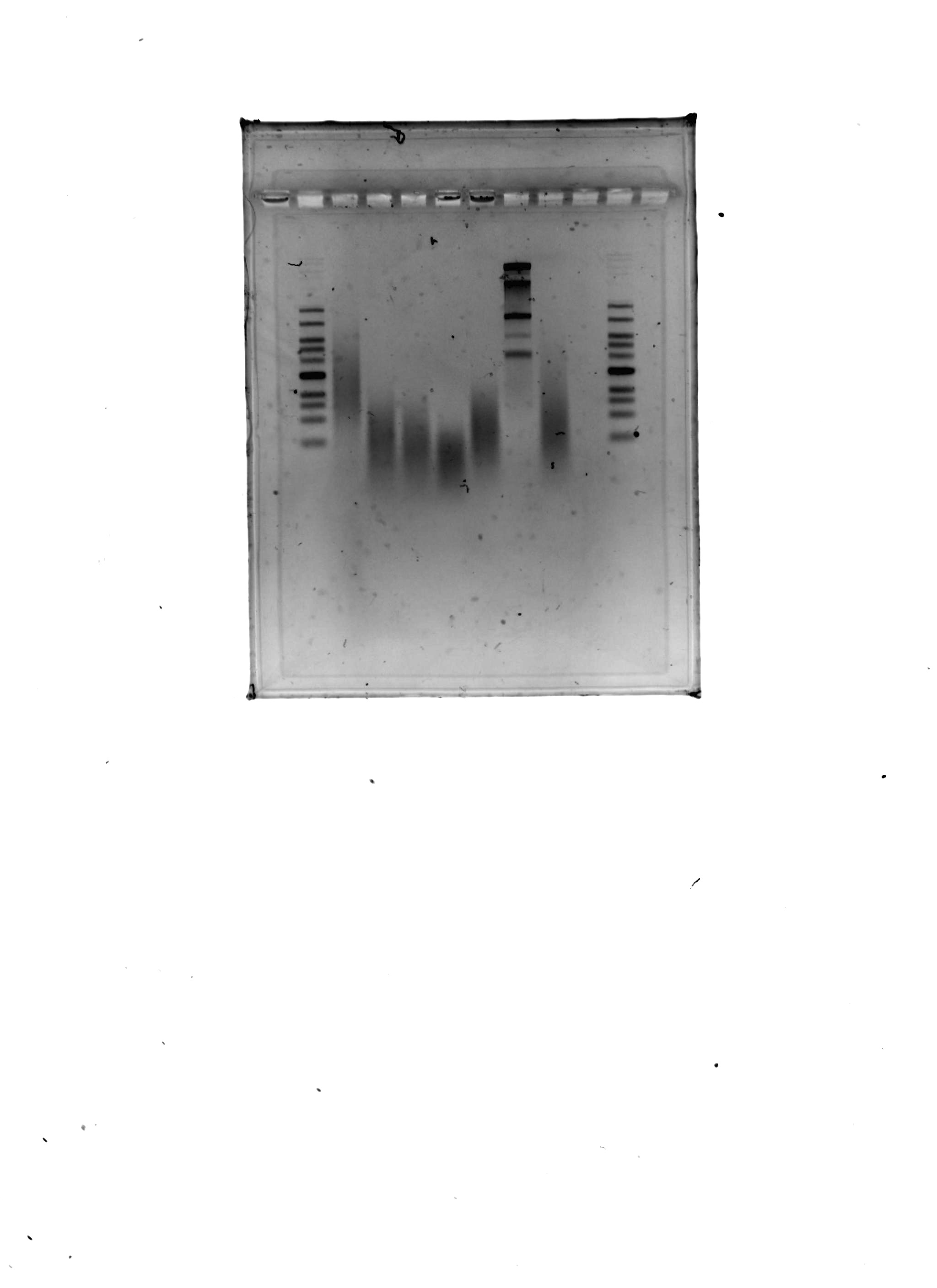

Supplement: Source data 1. [file elife-69544-data1.zip › eLife-source data/Figure 4-figure supplement1 source dataA.jpg]

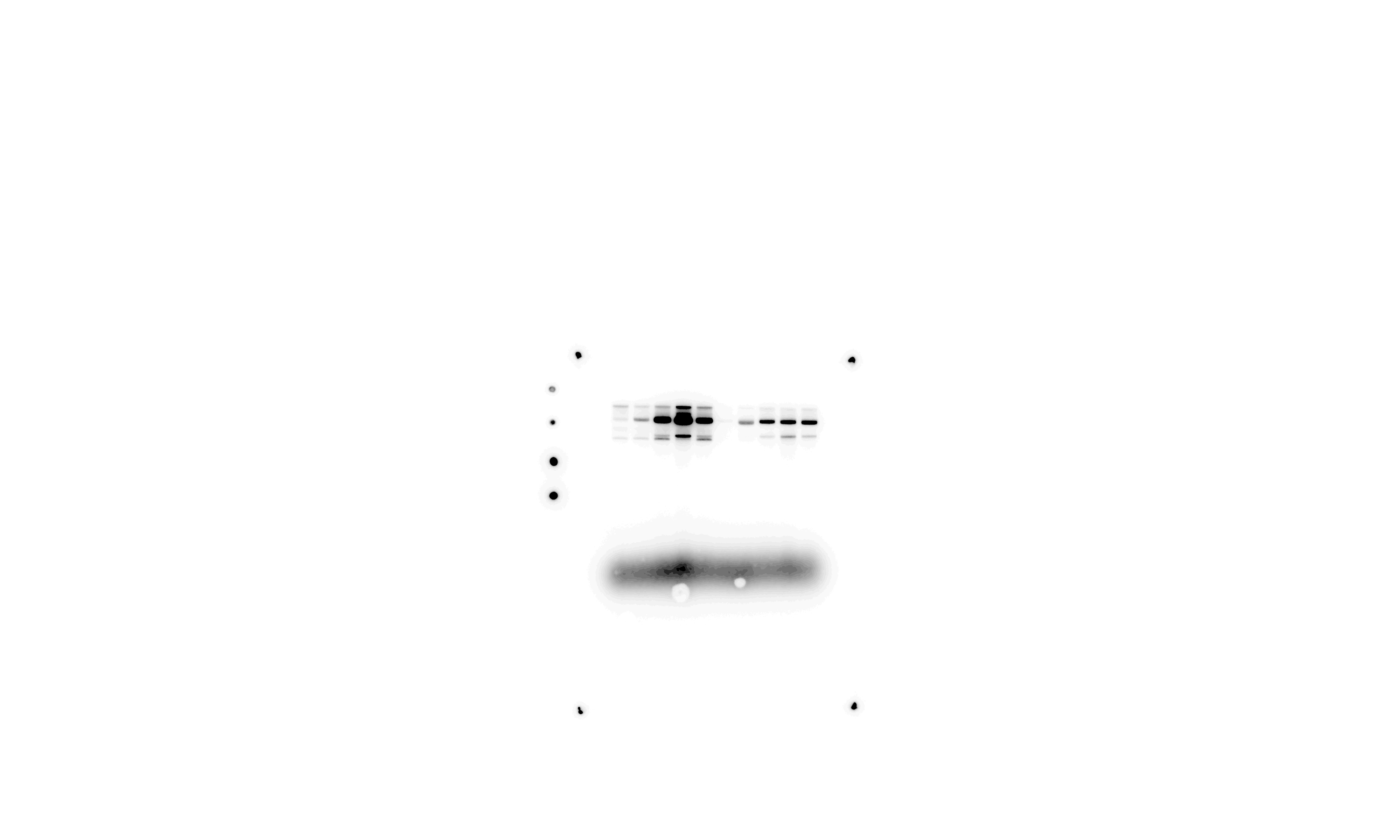

Supplement: Source data 1. [file elife-69544-data1.zip › eLife-source data/Figure 4-source dataA2.tif]

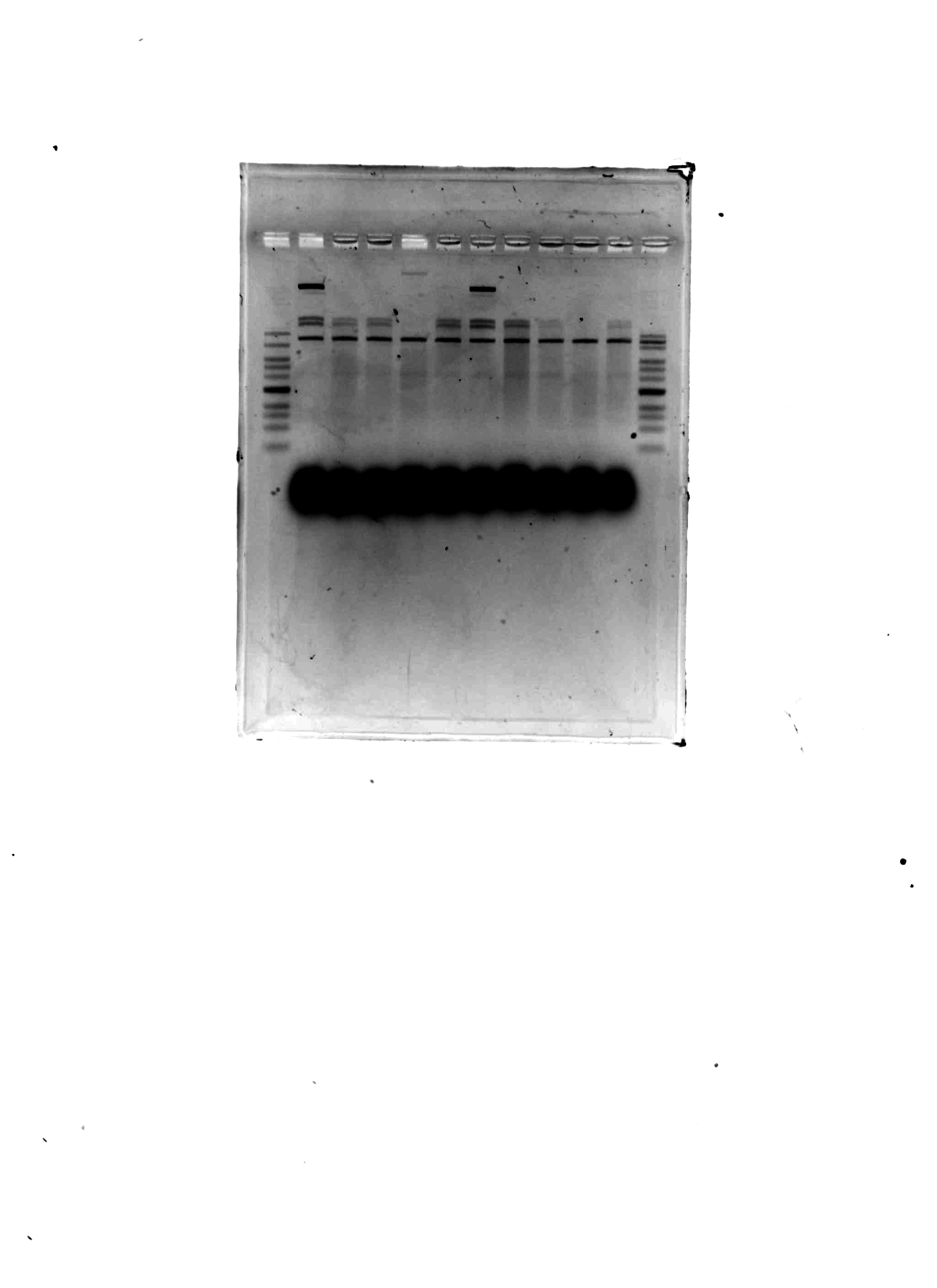

Supplement: Source data 1. [file elife-69544-data1.zip › eLife-source data/Figure 4-figure supplement2 source dataB1.jpg]

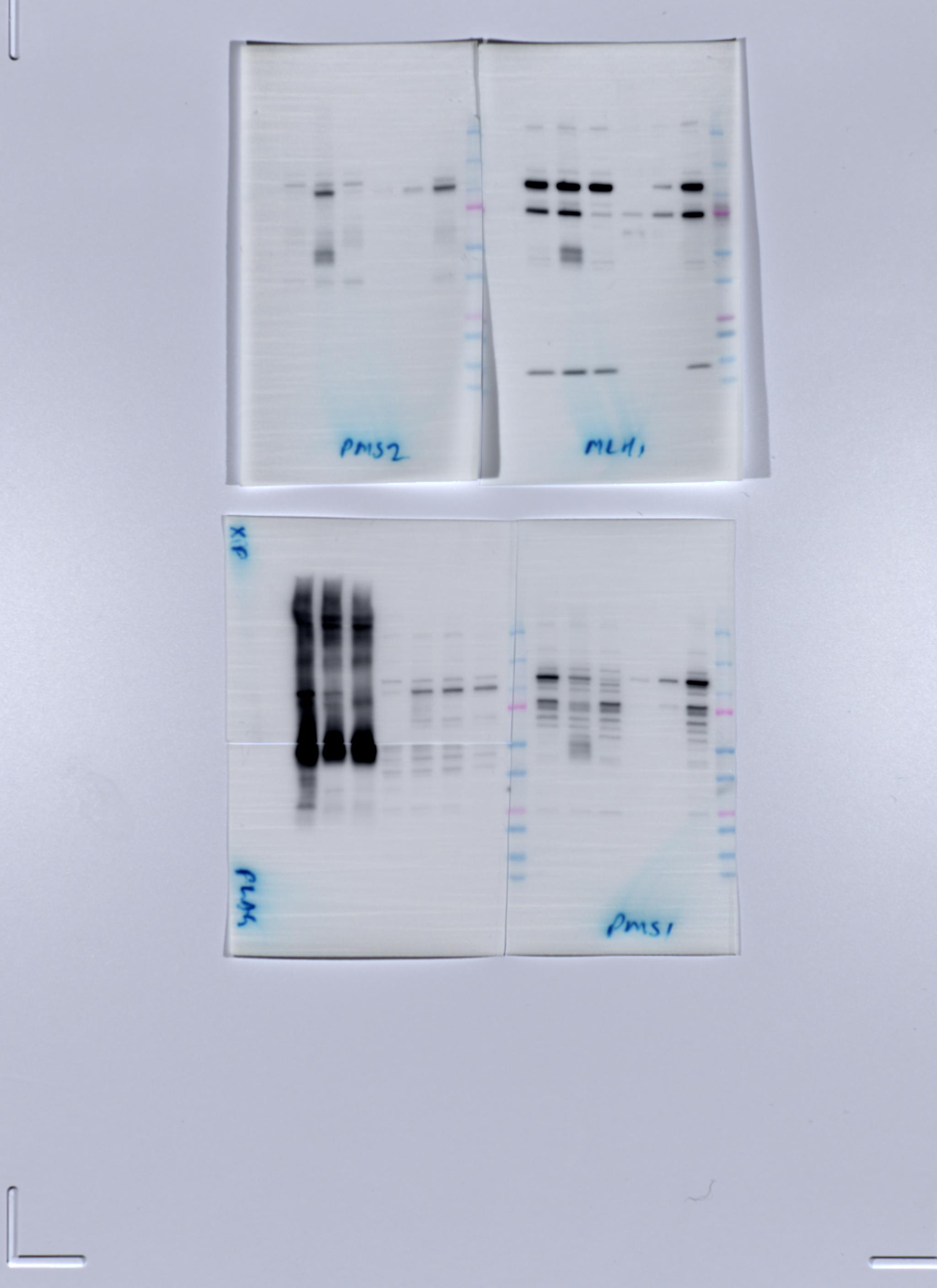

Supplement: Source data 1. [file elife-69544-data1.zip › eLife-source data/Figure 2-figure supplement1 source data.jpg]

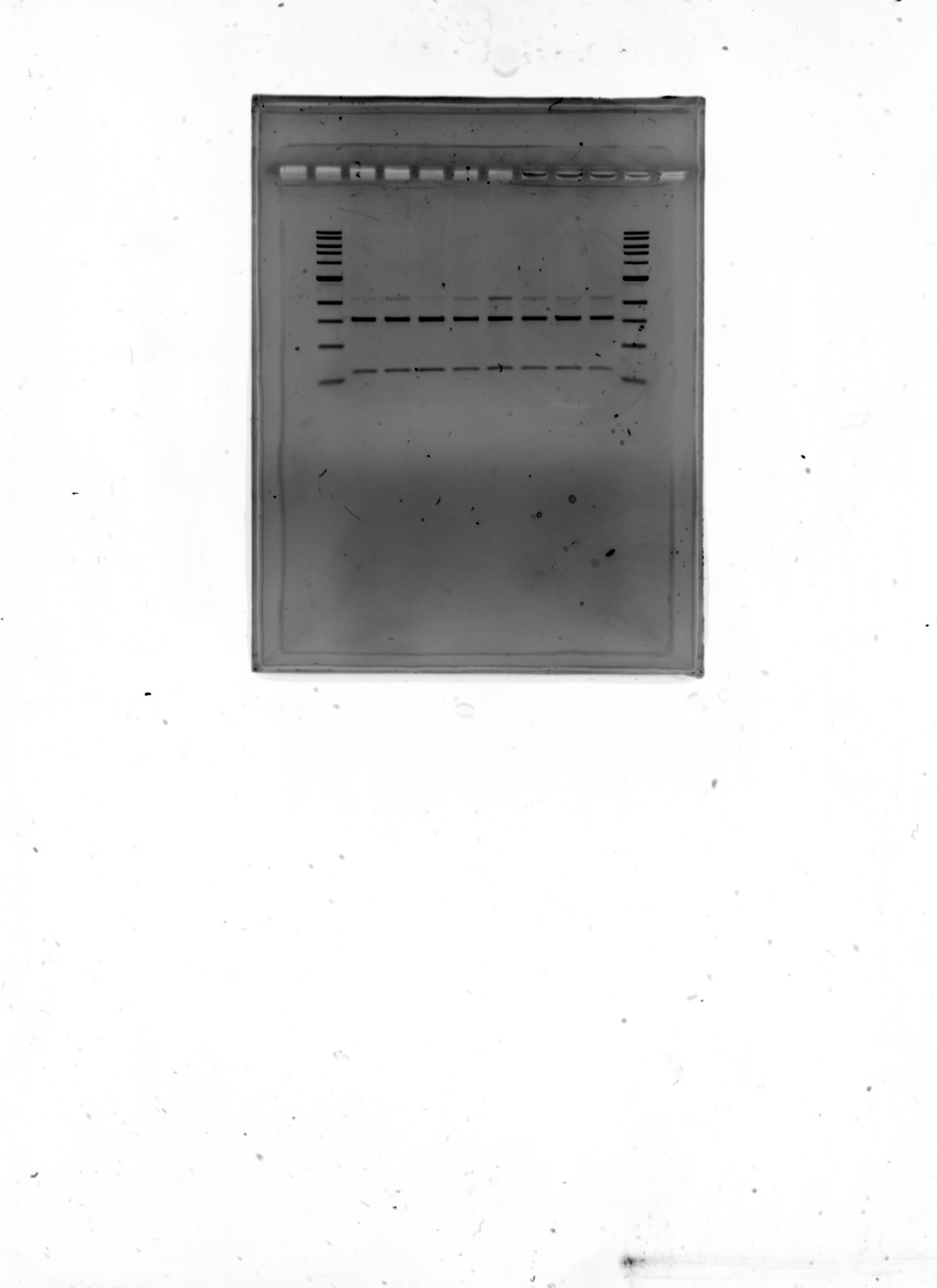

Supplement: Source data 1. [file elife-69544-data1.zip › eLife-source data/Figure 3-figure supplement1 source dataC1.jpg]

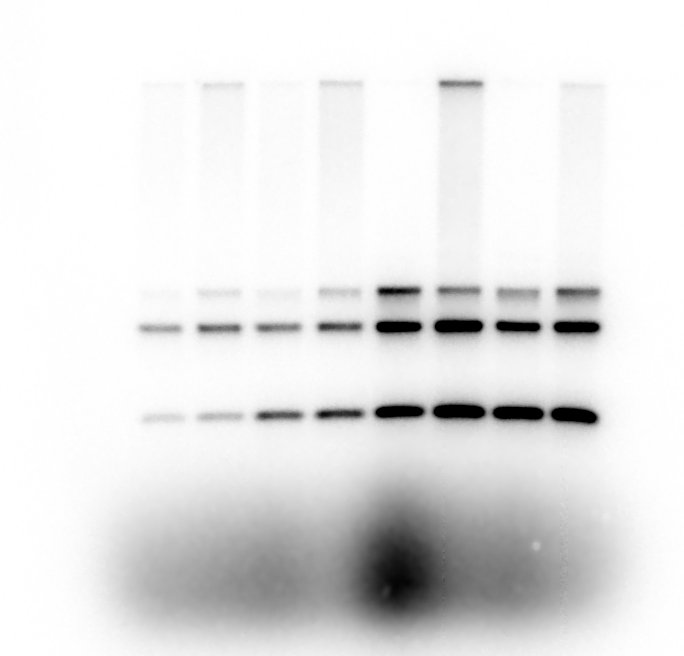

Supplement: Source data 1. [file elife-69544-data1.zip › eLife-source data/Figure 3-figure supplement1 source dataC2.jpg]

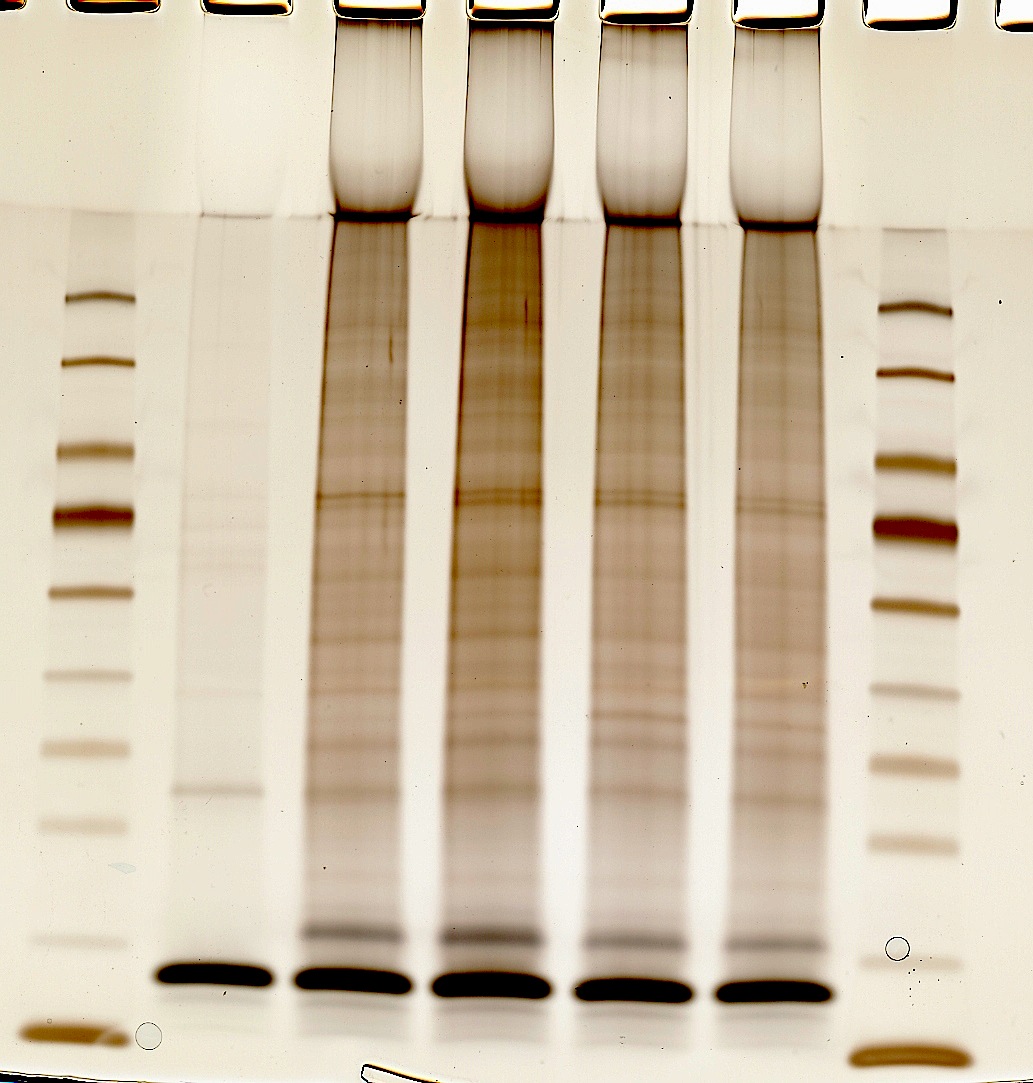

Supplement: Source data 1. [file elife-69544-data1.zip › eLife-source data/Figure 1-figure supplement1 source dataB.jpg]

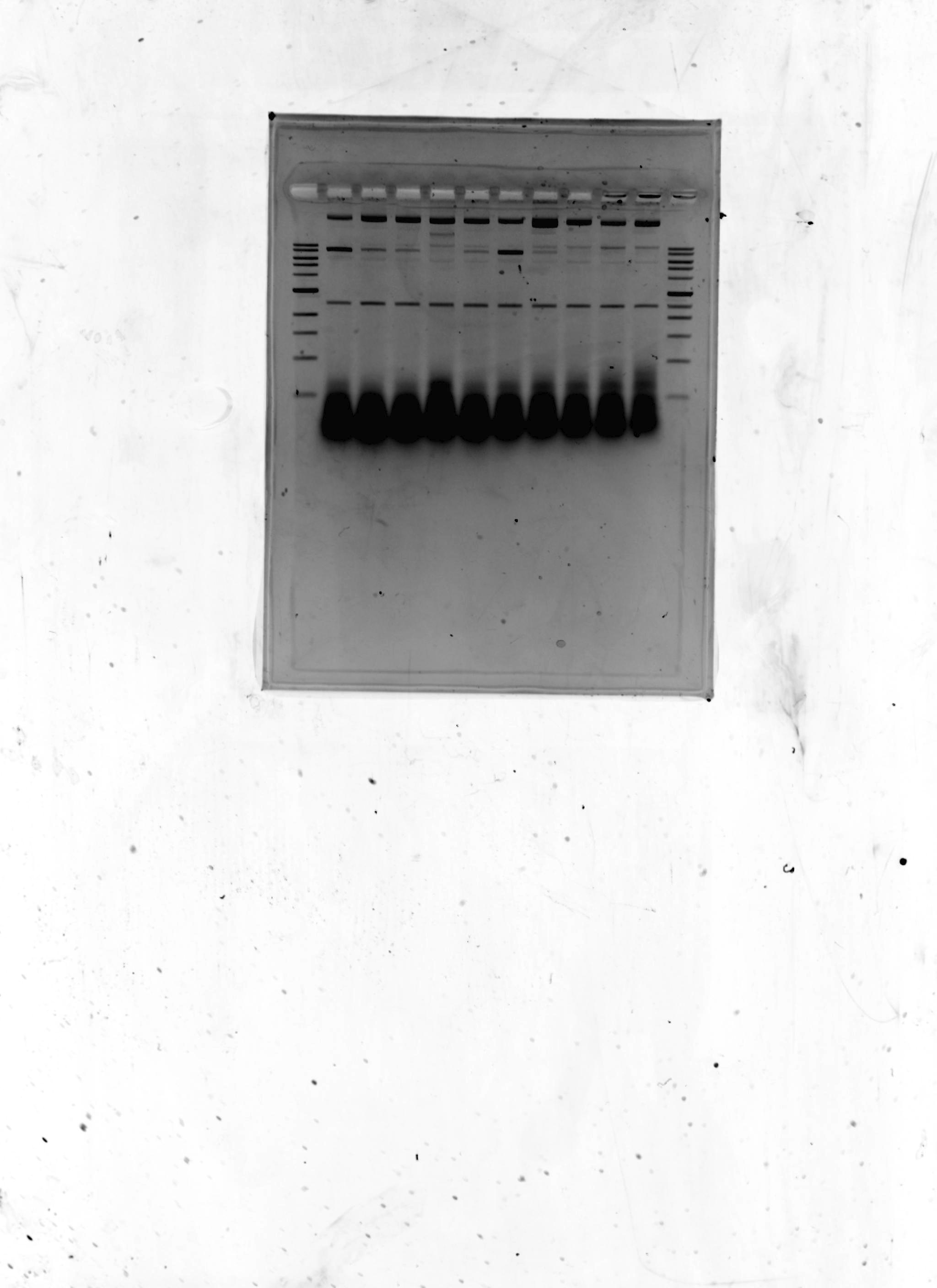

Supplement: Source data 1. [file elife-69544-data1.zip › eLife-source data/Figure 4-source dataA1.jpg]

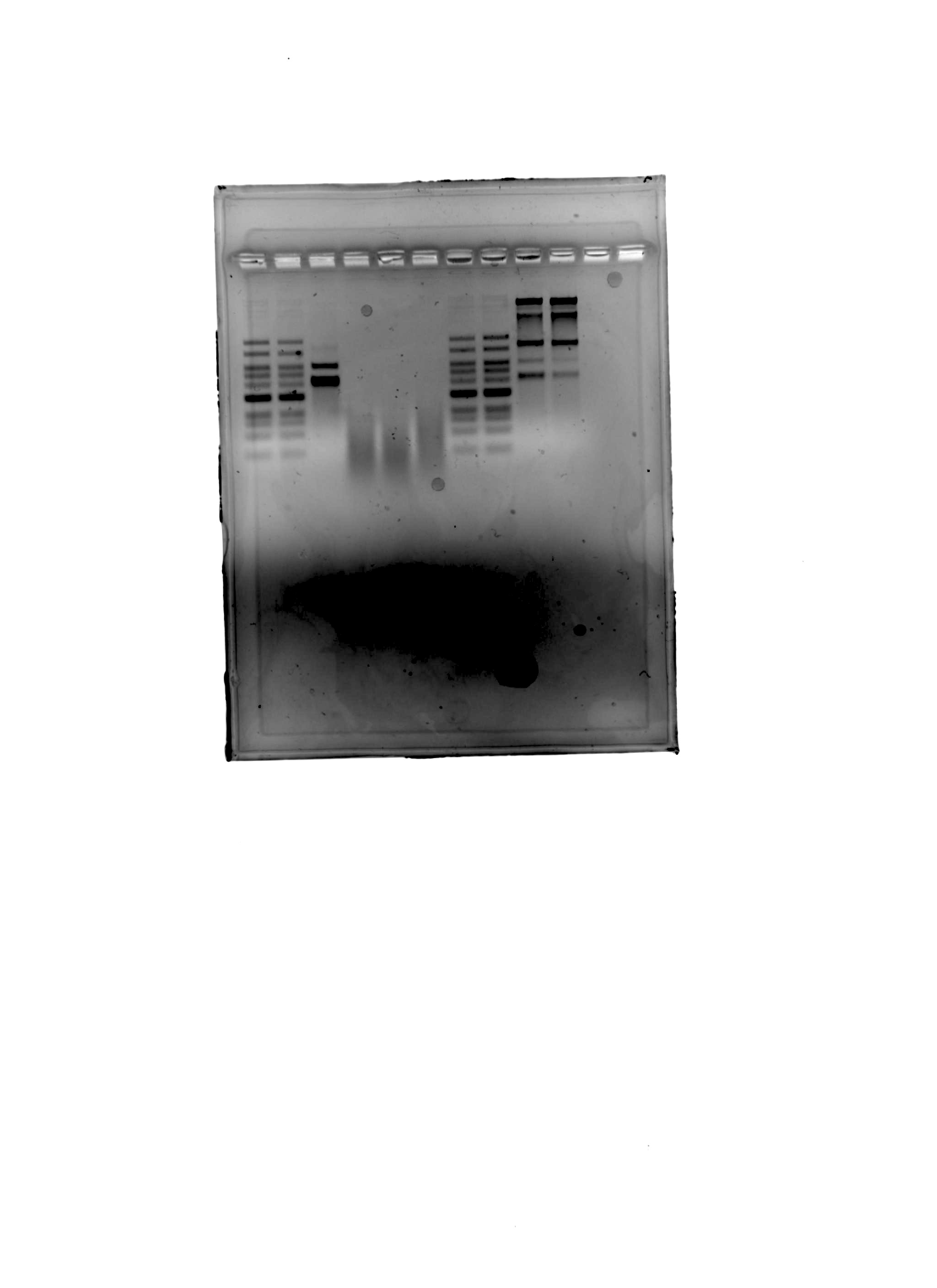

Supplement: Source data 1. [file elife-69544-data1.zip › eLife-source data/Figure 1-figure supplement1 source dataA.jpg]
